# Cardiac transthyretin amyloidosis ^99m^Tc-DPD SPECT correlates with strain echocardiography and biomarkers

**DOI:** 10.1007/s00259-020-05144-8

**Published:** 2020-12-26

**Authors:** Viktor Löfbacka, Jan Axelsson, Björn Pilebro, Ole B. Suhr, Per Lindqvist, Torbjörn Sundström

**Affiliations:** 1 Heart Centre, Clinical Physiology, Department of Surgical and Perioperative Sciences, Umeå University, Umeå, Sweden; 2 Radiation Physics, Department of Radiation Sciences, Umeå University, Umeå, Sweden; 3 Heart Centre, Cardiology, Department of Public Health and Clinical Medicine, Umeå University, Umeå, Sweden; 4 Diagnostic Radiology, Department of Radiation Sciences, Umeå University, Umeå, Sweden

**Keywords:** Amyloidosis-hereditary, Cardiomyopathy, Transthyretin amyloidosis, ^99m^Tc-DPD scintigraphy, 2D speckle tracking strain, SPECT

## Abstract

**Purpose:**

Hereditary transthyretin-amyloid amyloidosis (ATTRv) is an underdiagnosed condition commonly manifesting as congestive heart failure. Recently, scintigraphy utilizing DPD as a tracer was shown to identify ATTRv and wild-type ATTR cardiomyopathy. The aim of this study was to determine the value of quantified scintigraphy utilizing ^99m^Tc-3,3-diphosphono-1,2-propanodicarboxylic acid (DPD) single-photon emission computed tomography (SPECT)/CT, and to correlate its uptake with well-established cardiac functional parameters.

**Methods:**

Forty-eight patients with genetically verified ATTRv type-A fibril composition, positive ^99m^Tc-DPD SPECT/CT, were retrospectively analyzed. Manual mapping of volumes of interest (VOIs) on DPD SPECT/CT examinations was used to quantify heart uptake. DPD mean and maximum uptake together with a calculated DPD-based amyloid burden (DPD_load_) was correlated with echocardiographic strain values and cardiac biomarkers.

**Results:**

Statistically significant correlations were seen in VOIs between DPD uptakes and the corresponding echocardiographic strain values. Furthermore, DPD_load_ had a strong correlation with echocardiographic strain parameters and also correlated with biomarkers troponin T and logarithmic NT-ProBNP.

**Conclusions:**

In patients with ATTRv cardiomyopathy, DPD SPECT/CT measures the amyloid distribution and provides information on cardiac amyloid load. DPD amyloid load correlates with functional cardiac parameters.

## Introduction

Systemic amyloidosis is a group of diseases caused by the deposition of insoluble fibrils, known as amyloid fibrils, in the extracellular spaces of tissues. A wide range of organs can be affected by the diseases [1, 2]. Cardiac amyloidosis (CA) is a common manifestation of systemic amyloidosis, often representing with congestive heart failure and cardiac arrhythmias [3]. CA is an increasingly recognized condition and a more common condition than earlier perceived [4, 5].

One of the most common causes of CA is transthyretin (TTR) amyloid (ATTR) amyloidosis [6]. ATTR is a heterogeneous disease that can be divided into a hereditary form caused by mutations in the transthyretin-gene (ATTRv) and wild-type transthyretin amyloidosis (ATTRwt)—previously designated senile systemic amyloidosis [7]. It is of importance to identify ATTR-CA as conventional heart failure medication has no proven effect on prognosis and might even be harmful [8–10]. In addition, research of novel treatments is under development [11, 12].

Recently, nuclear medicine imaging by scintigraphy has become an important part of the diagnosis of ATTR CA. Various tracers, all regarded equally effective, have been employed including 99mTechnetium (Tc)-bisphosphonate derivatives (^99m^Tc pyrophosphate, ^99m^Tc-3,3-diphosphono-1,2-propanodicarboxylic acid [DPD], and ^99m^Tc hydroxymethylene-diphosphonate) [13–15]. DPD scintigraphy detects ATTR with type A fibril composition which is found in both ATTRwt and the vast majority of ATTRv mutations [16–18].

Currently, DPD scintigraphy has mainly been used for the diagnosis of ATTR CA based on the visual Perugini score [19]. While other biomarkers predict survival, a non-zero Perugini score fails to predict survival [20]. Other methods based on planar images have also been investigated [20–22]. Recent studies have suggested that DPD planar scintigraphy correlates with cardiac biomarkers and echocardiographic findings [23, 24]. DPD single-photon emission computed tomography (SPECT) was shown to correlate to cardiac magnetic resonance and Perugini score [25, 26]. It has recently been suggested that DPD SPECT/CT has a better diagnostic performance than planar quantification techniques [27–29].

Echocardiography and echocardiographic strain analysis are widely studied, important diagnostic and prognostic tools for CA [30–33]. The new treatments have increased demand for imaging techniques that can evaluate the cardiac amyloid burden and function to assess the efficacy of treatment [24, 34, 35]. It is plausible that in addition to echocardiography, quantitative DPD might have an important role in the evaluation of upcoming treatments.

The aim of this study was to use DPD SPECT/CT to quantify DPD uptake in the ventricular walls of the heart in type A fibril ATTRv patients and relate the outcome to cardiac function assessed by echocardiographic strain, troponin T and NT-ProBNP.

## Materials and methods

### Patient selection

In this retrospective study, 48 patients fulfilled the following inclusion criteria: (1) genetically verified ATTRv amyloidosis, (2) an abdominal tissue biopsy displaying type-A fibril composition, (3) a positive DPD examination, (4) no coronary artery disease, and (5) echocardiographic examinations within 1 year of the DPD examination. All patients were examined between 2012 and 2015 at the Swedish Centre for Hereditary Amyloidosis in Umeå, Sweden.

### Data collection

Clinical records were examined for information on mortality, presence of hypertension, atrial fibrillation, presence of a pacemaker, and NT-ProBNP and troponin T levels. NT-ProBNP and troponin T levels were only included in the analysis if tests were done within 30 days of DPD examinations. Data on fibril typing, status of liver transplantation, and visual scoring grade on DPD uptake according to Perugini grading was also acquired; the latter was performed in a clinical setting.

### DPD scintigraphy

All patients were investigated with an Infinia Hawkeye hybrid single-photon emission computed tomography gamma camera (SPECT-CT) (General Electric Medical Systems, Milwaukee, WI, USA,) with a low-energy high-resolution collimator. An intravenous injection of 740 (± 52) MBq DPD was performed 3 h prior to the acquisition of a 256 × 1024 matrix whole-body planar image, followed by a non-contrast, low-dose CT scan. Following the CT, a SPECT acquisition was performed in 60 projections, 360° camera rotation, iteratively reconstructed to a 128 × 128 matrix (OSEM, 3 iterations, 10 subsets) with scatter and CT-based attenuation correction and Butterworth filter with cut-off frequency 0.45, order 7. Reconstruction of SPECT images was performed on the Xeleris workstation (GE Healthcare, Waukesha, WI, USA).

Images were reformatted from a transaxial view to orthogonal short- and long-axis views of the heart using ImageJ (ImageJ 1.44o, Wayne Rasband, National Institutes of Health, USA) software. This was performed to achieve a robust volume of interest (VOI) delineation of the cardiac anatomical structures. To achieve a DPD quantification, VOI values were first decay-corrected. Then, a standardized uptake value was calculated by dividing the decay-corrected VOI values with the injected dose per kilogram bodyweight, referred to as DPD uptake. The unit of the standardized uptake value in DPD_mean_ and DPD_max_ is grams per milliliter with an unknown scale factor, because the scanner and the well counter were not cross-calibrated.

**Fig. 1:**
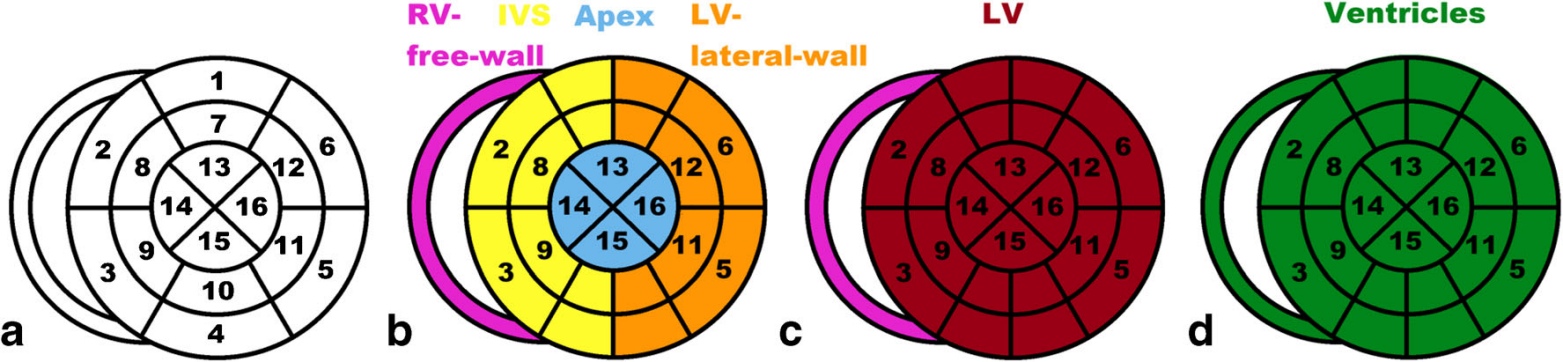
Different regions are presented in the following colours: purple—right ventricular free wall (RV free wall); yellow—interventricular septum (IVS); blue—apex of the heart (Apex); orange—left ventricular lateral wall (LV lateral wall). The definition of these follows stepwise. A. Description of the echocardiographic 16-segment short axis bulls eye model. B. Regions used in the study. The anterior segments [1, 7] and the posterior segments [4, 15] were split in half and joined with their respective side. C. Regions used to compare uptake against echocardiographic strain: red—left ventricle (LV), purple—right ventricular free wall (RV free wall). D. Region used to compare uptake against cardiac biomarkers: green—heart ventricles (whole heart)

**Fig. 2:**
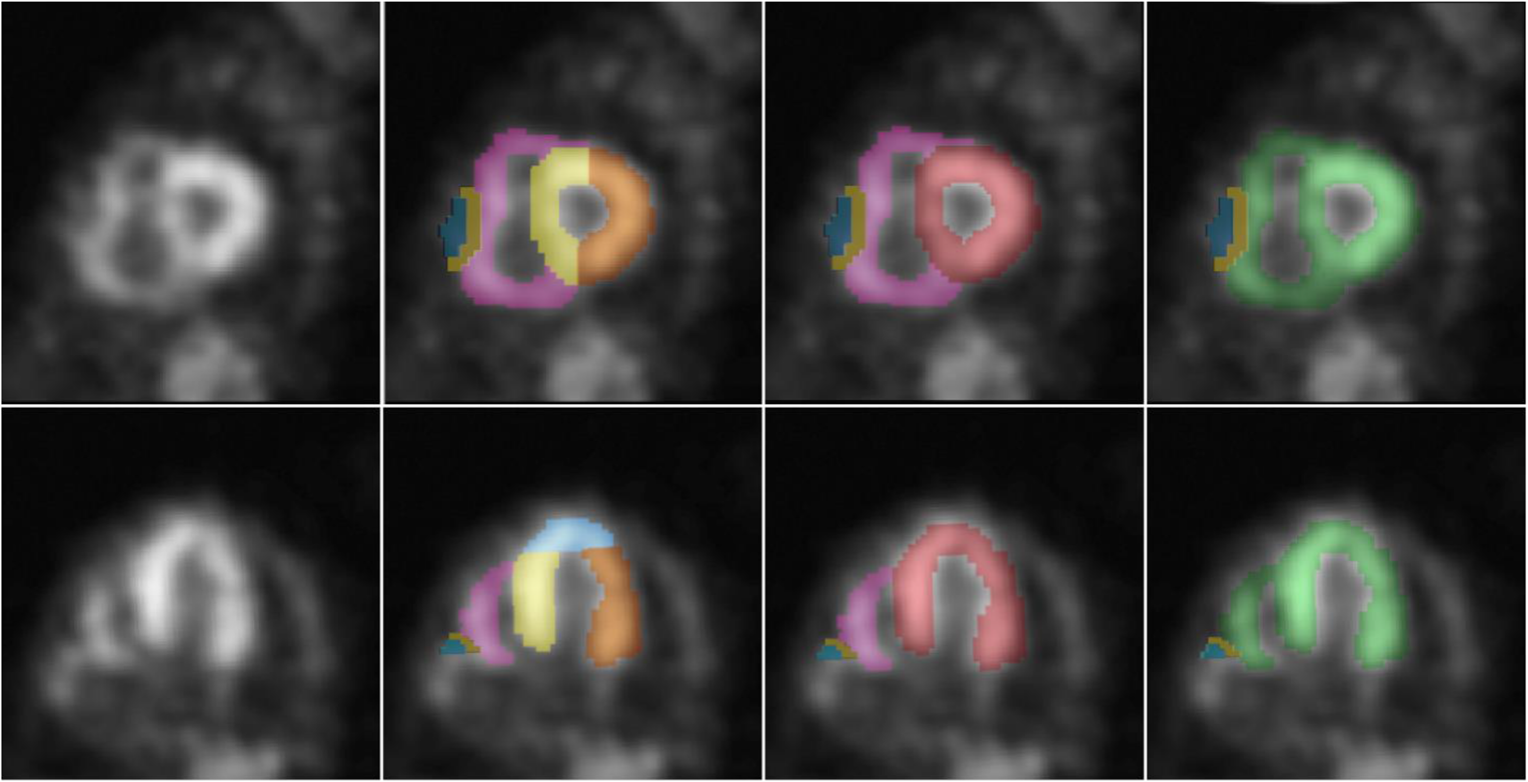
DPD-SPECT slices in apical short axis view (top panels) and apical long axis view (bottom panels) demonstrating volumes of interest (VOIs) mapping and sternum isolation from the right ventricular wall for minimizing the sternal activity spill-over. Different VOIs are presented in colours as defined in Fig. 1. Purple—right ventricular free wall (RV-free-wall); yellow—interventricular septum (IVS); blue—apex of the heart (Apex); orange—left ventricular lateral wall (LV lateral wall); red—left ventricle (LV); green—heart ventricles (whole heart); mustard yellow—boarder around sternum; turquoise—sternum spill over

A standardized 16-segment echocardiographic short axis model described by the American Society of Echocardiography (ASE) [36] was used as a starting point to define regions to quantify. Because coronary perfusion territories were not relevant for the study, the model was simplified. Mapping modifications were conducted as described in Fig. 1. The mapped heart structures included LV lateral wall, interventricular septum (IVS), apex of the heart (Apex), and RV free wall (Fig. 2). RV free wall was mapped like the other VOIs, but in some cases, parts of the RV free wall directly adjacent to sternum were excluded from the VOI to avoid spill-over from the sternum. This was carried out by identifying and adding a border around the sternum, to minimize the influence of sternum spill over (Fig. 2). All heart-related quantification mappings were done in the cardiac short axis projections.

A manual VOI delineation of different anatomical regions was done in the SPECT images by one examiner (co-author VL), under guidance of an experienced specialist in radiology and a nuclear medicine specialist (co-author TS). The intention was to achieve near anatomical VOIs without having access to a contrast enhanced CT.

To achieve unbiased VOIs more accurately representing the DPD uptake, a segmentation volume with a threshold of 42% (V_42%_) of the max uptake [37, 38] was used. For each segmented VOI, the mean voxel DPD uptake (DPD_mean_) and the maximum voxel DPD uptake (DPD_max_) were calculated. The parameter DPD_load_, representing a DPD-based amyloid burden, was calculated as DPD_load_ = DPD_mean_* V_42%_. The intention of the DPD_load_ is to be a compound measure, which takes both the pathological volume and the DPD uptake into account.

We also created two larger merged VOIs (Fig. 2) for a statistically more robust comparison to cardiac biomarkers. Left ventricle (LV) was created by merging LV lateral wall, IVS, and apex. A VOI consisting of all mapped heart regions, called “ventricles”, was created by adding the RV free wall to LV. Echocardiographic LV global longitudinal strain (GLS) was compared to DPD uptake in the LV VOI.

Imlook4d free software (“https://sites.google.com/site/imlook4d/”) and MATLAB (Release 2018b, The MathWorks Inc., Natick, MA, USA) were used for the DPD VOI analysis.

### Echocardiography

Patients underwent a comprehensive echocardiographic examination using GE Vivid 7 or E9 (GE Vingmed Ultrasound, Horten, Norway). The echocardiographic analysis was made offline using commercially available software packages like Echopac PC version 113 (GE Healthcare, Horten, Norway). Analysis and regions of interest (ROI) delineation were performed by one operator (co-author VL), under the supervision and support of an experienced echocardiographer (co-author PL).

**Fig. 3:**
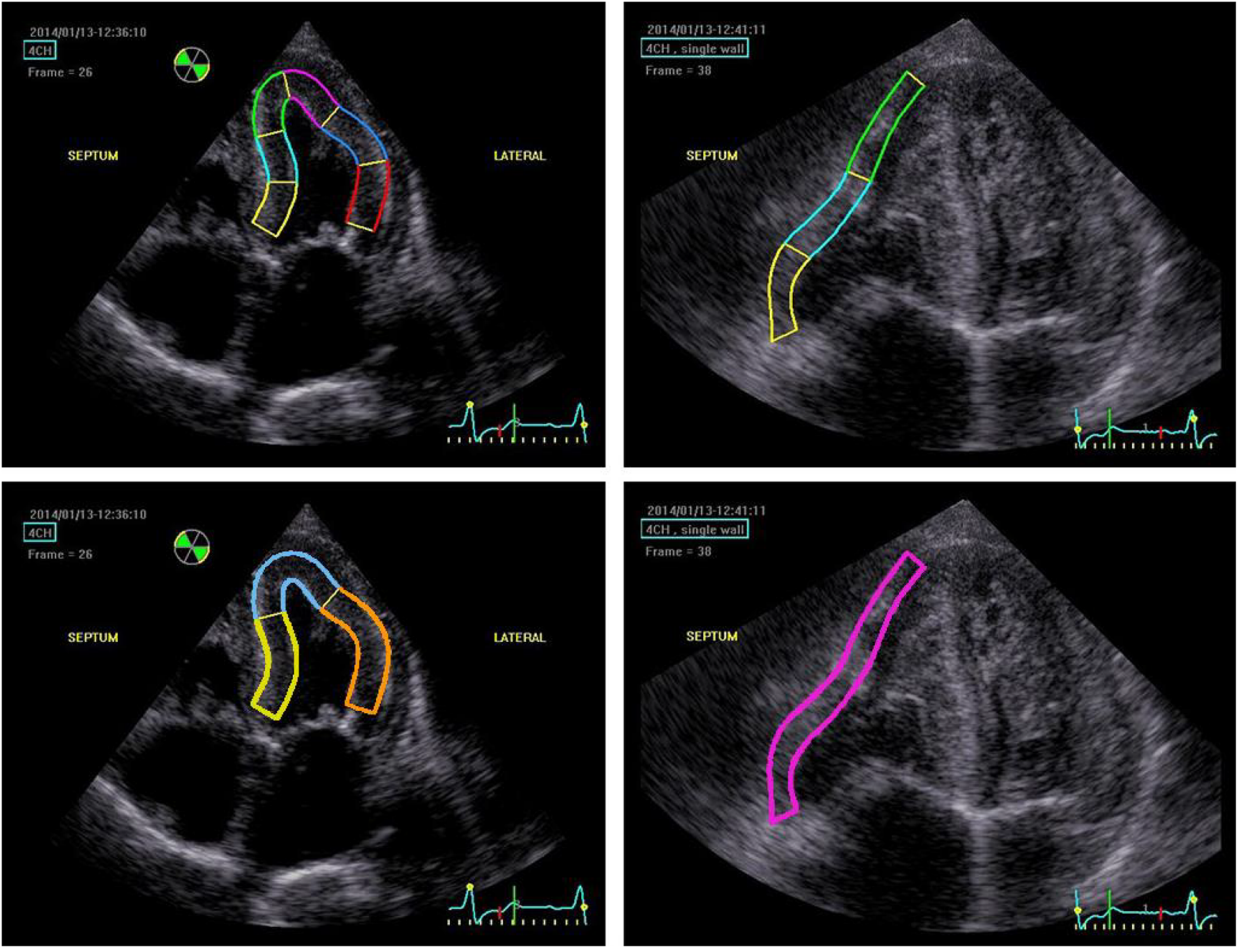
Images of echocardiographic speckle tracking strain analysis in apical four-chamber view projections. Top panels show full wall regions of interest (ROIs) mappings for left ventricular global longitudinal strain (LV) (left) and right ventricular free wall strain (RV free wall) (right). Bottom panels show how different echocardiographic ROIs match the DPD scintigraphic volumes of interest (VOIs) described in Fig. 2

The speckle tracking strain analyses were conducted according to ASE guidelines from the apical four-chamber view, during systole [36]. End systole was defined by mitral valve motion from anatomic M-mode or from T-wave from the superimposed electrocardiography. RV free wall strain and GLS analysis of LV were done manually by tracing the endocardial borders. ROIs were automatically defined by the software. Papillary muscles were excluded in all strain measures as per the ASE guidelines. When needed, segments were manually adjusted after the automatic ROI generation, and insufficient ROIs were excluded. Due to thickened ventricular walls, ROI analysis on the full wall thickness was not always possible and was often adjusted manually. Matching of ROI segments with DPD VOIs is described in Figs. 1, 2, and 3. For normal values, values from the European Association Cardiovascular Imaging were used [39].

### Apical sparing

In echocardiography, a relative apical sparing was calculated according to the following formula: average apical longitudinal strain/(average basal longitudinal strain + average mid-longitudinal strain), with a cut-off of 1 [40]. To compare uptake in the left side of the heart, DPD_mean_ in LV lateral wall and IVS was compared to DPD_mean_ in the apex.

### Statistics

Statistical analyses were done using SPSS®, version 26 (IBM). Pearson’s correlations were used to express correlations, and *p* values were calculated using a two-tailed *t* test. A *p* value of < 0.05 was considered statistically significant. ANOVA by Kruskal-Wallis was used to compare the relationships between the Perugini scale and DPD-load.

## Results

### Patient characteristics

Clinical records received for patients are summarized in Table 1. A total of eight different ATTR mutations were present. The most common mutation was Val30Met found in 39 of the 48 patients (81%). Two patients (4%) had a Ala45Gly mutation and another two had a Ala97Ser mutation. The following five mutations Ala45Ser, Glu54Leu, His88Arg, Val30Leu, and Val122Ile were found in one patient (2%) each. At the time of the summation of the data (mean 59 months after DPD examination), twelve of the patients (25%) had died (Table 1). Thirty-five patients (73%) had a Perugini grade 3, ten (21%) grade 2, and only three (6%) had grade 1.

**Table 1:** Clinical and demographic characteristics of included patients

	*N*	%	Mean	Min	Max	SD
Gender (male)	35/48	72.9				
Mortality	12/48	25.0				
Age (years)	48/48	100.0	69	42	83	7.0
Age at death (years)	12/48	25.0	73	42	85	10.7
Atrial fibrillation	12/48	25.0				
Pacemaker	8/48	16.7				
Hypertension	9/48	18.8				
Liver transplantation	4/44	8.3				
Troponin T (ng/l)	36/48	75.0	28.8	4	75	18.0
NT-ProBNP (ng/l)	40/48	83.3	1588.5	82	12,000	2349.6
log_10_(NT-ProBNP)	40/48	83.3	2.8	1,9	4,1	0.6
Height (cm)	45/48	93.8	175.3	157.0	190.0	8.5
Weight (kg)	48/48	100.0	74.6	50.0	115.0	14.8
BMI (kg/m^2^)	45/48	93.8	24.3	18.1	34.4	4.0

### DPD analysis

Uptake values for DPD VOIs are presented in Table 2. The highest DPD_mean_ and DPD_max_ were seen in IVS follow by LV lateral wall and apex. There was a strong relationship between DPD_mean_ and DPD_max_ values in all VOIs (*p* < 0.001) (data not shown).

**Table 2:** ^99m^Tc-DPD measurements for different volumes of interest, defined in Fig. 2

	Mean	Min	Max	SD
DPD_mean_				
LV lateral wall	43.4	15.34	83.6	17.5
IVS	54.6	16.6	99.0	20.1
Apex	36.5	10.9	81.8	16.0
RV free wall	30.2	12.4	59.2	10.6
LV	51.4	15.9	92.7	18.9
Ventricles	50.9	15.7	90.9	18.7
DPD_max_				
LV lateral wall	67.8	25.6	131.7	26.0
IVS	87.5	26.4	158.0	32.5
Apex	57.6	17.5	121.9	24.3
RV free wall	53.9	22.7	107.3	18.6
LV	87.7	26.4	158.0	32.3
Ventricles	87.7	26.4	158.0	32.3
DPD_load_				
LV lateral wall	4448.0	221.8	10,629.2	2595.8
IVS	5099.0	1196.5	12,173.6	2685.9
Apex	1100.2	104.8	2956.1	700.8
RV free wall	2574.1	200.9	6864.6	1476.1
LV	9429.5	121.3	23,008.1	5252.1
Ventricles	10,144.4	1214.0	24,997.0	5688.7

**Fig. 4:**
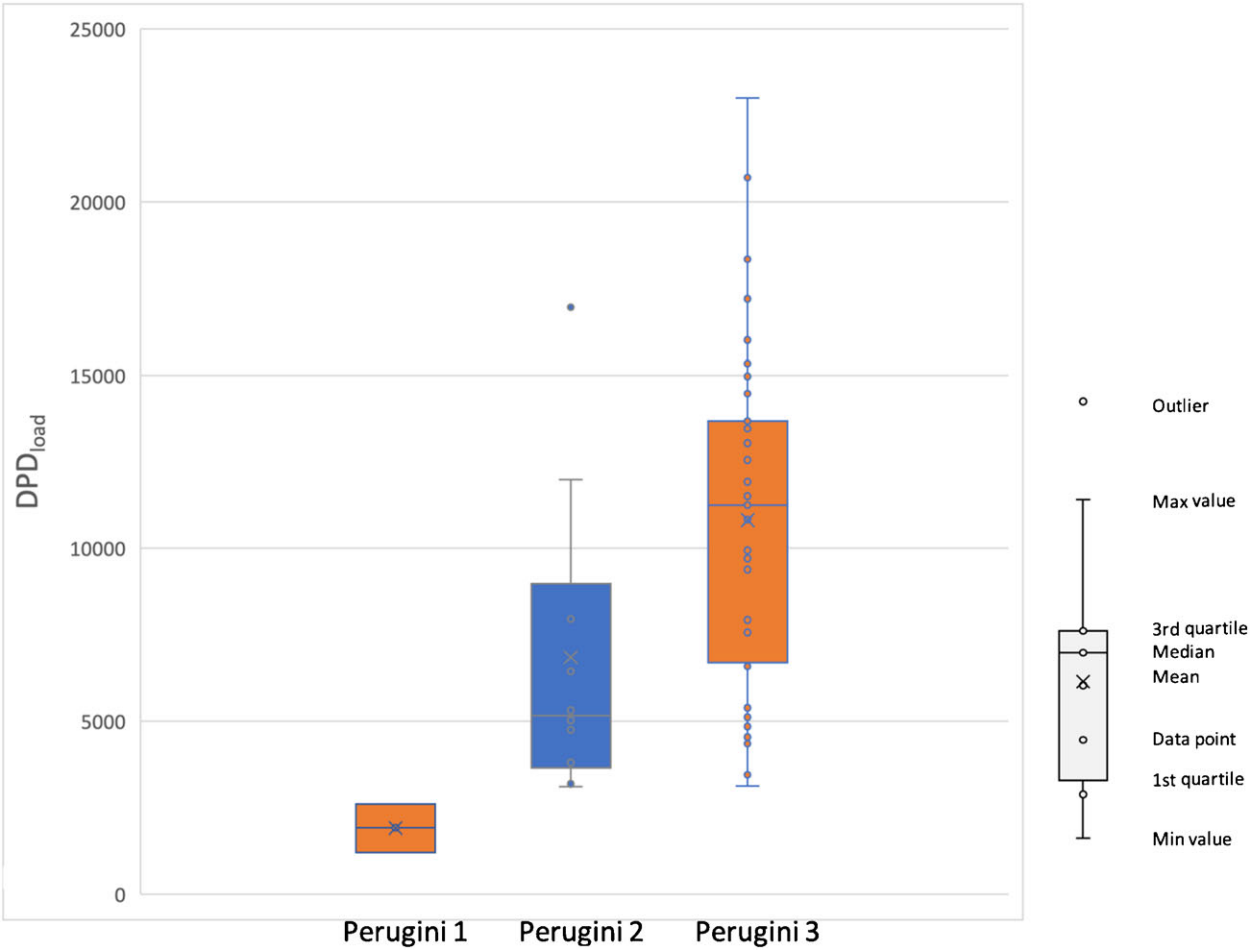
Boxplot showing distribution of LV DPD_load_ for different Perugini grades

For both regions, LV and ventricles, there was a significant correlation (*p* = 0.001) between DPD_load_ and Perugini grade. When comparing the DPD_load_ distributions in these regions for different Perugini grades, the difference in DPD_load_ was significant between Perugini grades 1–3 and 2–3. For Perugini grade 1–2, the difference in DPD_load_ was not significant but there were only 3 patients with Perugini score 1. Although the distributions of DPD_load_ for patients with Perugini scores 2 and 3 were significantly different, the DPD_load_ values displayed a high degree of overlap (Fig. 4).

### Echocardiographic strain analysis

Apical four-chamber view projections were sufficient for echocardiographic speckle-tracking strain analysis of LV GLS in all cases. Thirty-three patients (69%) exhibited impaired LV GLS (values below − 20%). The image quality to measure RV free wall strain was sufficient in thirty-four patients (71%); of these, 21 patients (62%) showed impaired RV free wall strain (values below − 23%). Echocardiographic strain values, expressed as a negative percentage, are summarized in Table 3.

**Table 3:** Echocardiographic strain values for different regions of interest, defined in Fig. 3. The unit of strain is %

	*N*	Mean	Min	Max	SD
LV lateral wall	48	− 14.1	− 27.0	− 2.0	5.5
IVS	48	− 11.6	− 22.5	− 0.50	5.3
Apex	48	− 25.5	− 43.5	− 11.0	7.5
RV free wall	34	− 20.2	− 33.7	− 7.3	6.9
LV	48	− 17.0	− 28.3	− 6.7	5.1

### DPD relation to ATTR

When comparing DPD_mean_ to echocardiographic strain values, statistically significant correlations were seen for all VOIs (*p* = 0.049 for RV free wall, *p* ≤ 0.001 for all remaining regions). No statistically significant correlations were found toward any biomarkers. When comparing DPD_max_ to echocardiographic strain, statistically significant correlations were found in the VOIs for the left side of the heart (LV lateral wall, IVS, apex, and LV). No correlations were found between strain and RV free wall or with any of the biomarkers.

**Fig. 5:**
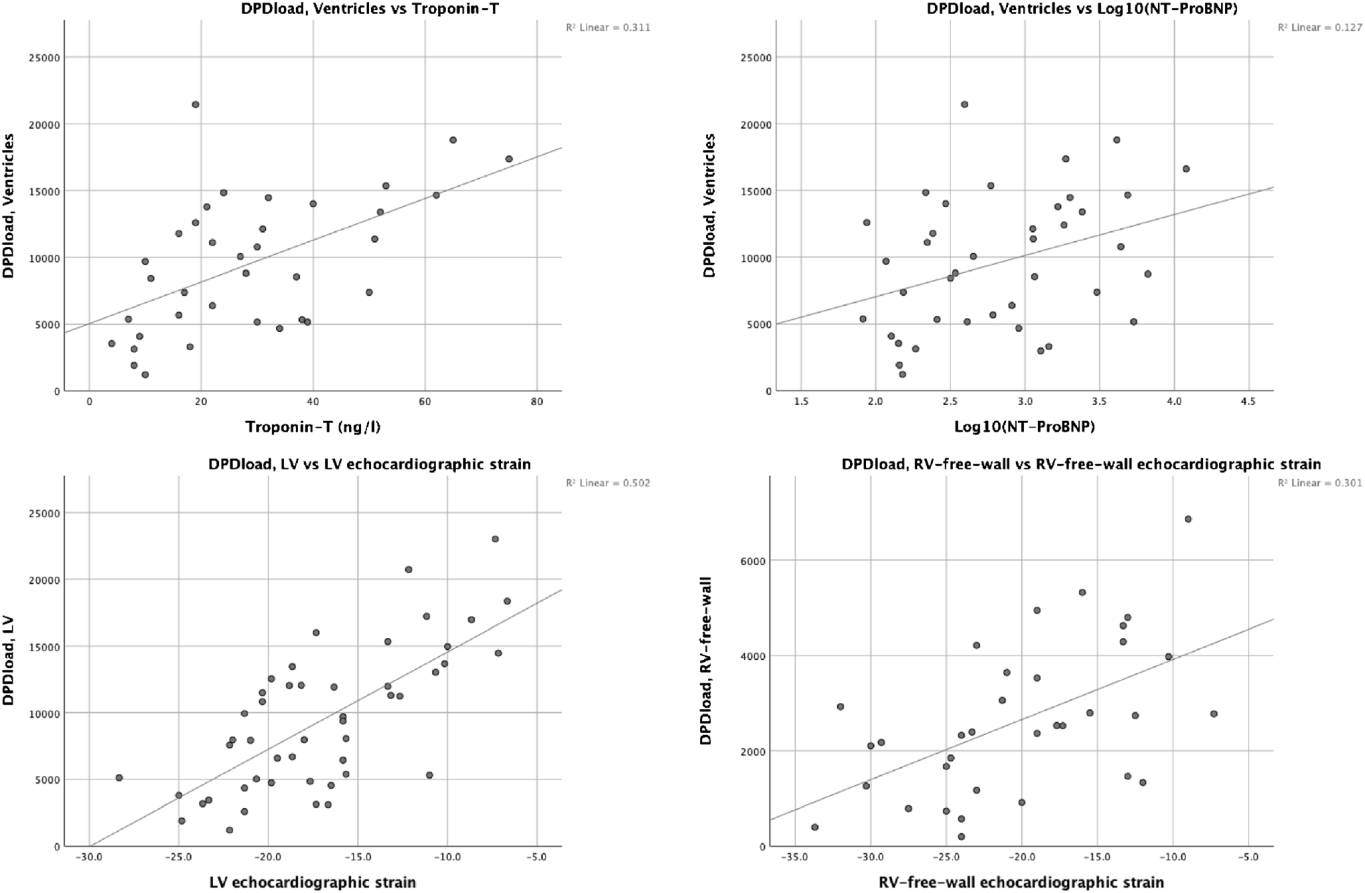
Top panels: scatterplots demonstrating ventricles DPD_load_ correlation to troponin T and log_10_(NT-ProBNP). Bottom panels; DPD_load_ correlation to echocardiographic strain in left ventricle (LV) and right ventricular free wall

DPD_load_ correlated significantly with all echocardiographic strain parameters, *p* ≤ 0.001 in all regions, as well as with the biomarkers troponin T (*p* < 0.001) and log_10_(NT-ProBNP) (*p* = 0.024) (Table 4). Scatterplots showing DPD_load_ correlations with LV, RV free wall strain, troponin T, and logarithmic NT-ProBNP are shown in Fig. 5.

**Table 4:** Table showing DPD correlations to their corresponding echocardiographic strain values and cardiac biomarkers

		Correlation	*R*	*p*
DPD_load_				
Correlation with DPD_load_ to echo strain and cardiac biomarkers.		LV lateral wall	0.572	< 0.001
		IVS	0.716	< 0.001
		Apex	0.531	< 0.001
		RV free wall	0.549	0.001
		LV	0.708	< 0.001
*For the three biomarkers, the ventricles DPD_load_ was used.		Troponin T*	0.558	< 0.001
		NT-ProBNP*	0.304	0.056
		log_10_(NT-ProBNP)*	0.356	0.024
DPD_mean_				
Correlation with DPD_mean_ to echo strain and cardiac biomarkers.		LV lateral wall	0.528	< 0.001
		IVS	0.495	< 0.001
		Apex	0.477	0.001
		RV free wall	0.340	0.049
		LV	0.572	< 0.001
*For the three biomarkers, the ventricles DPD_mean_ was used.		Troponin T*	0.303	0.072
		NT-ProBNP*	0.072	0.659
		log_10_(NT-ProBNP)*	0.058	0.723
DPD_max_				
Correlation with DPD_max_ to echo strain and cardiac biomarkers.		LV lateral wall	0.511	< 0.001
		IVS	0.478	0.001
		Apex	0.493	< 0.001
		RV free wall	0.335	0.053
		LV	0.536	< 0.001
* For the three biomarkers, the ventricles DPD_max_ was used.		Troponin T*	0.304	0.071
		NT-ProBNP*	0.032	0.847
		log_10_(NT-ProBNP)*	0.030	0.854

Analyzing Perugini scores towards the above biomarkers, the only biomarker that showed any significance was troponin from grade 1 to 3 (*p* = 0.03).

### Apical sparing pattern

When comparing strain deformation in echocardiography, higher deformation was found in the apex compared to the rest of the LV (Table 3). Using echocardiographic strain analysis, 24 patients (50%) showed a relative apical sparing. When comparing DPD_mean_ in the left side of the heart, the apex displayed significantly lower DPD_mean,_ compared to DPD_mean_ in both the LV lateral wall and IVS. Thus, a similar apical sparing pattern was observed in both DPD and echocardiographic strain analysis.

## Discussion

The main finding in this study was that DPD SPECT/CT measures ATTRv amyloid distribution and provides information on cardiac amyloid load. Furthermore, DPD amyloid uptake correlates with echocardiographic strain measures and cardiac biomarkers.

The mechanism behind DPD uptake in CA is still unclear; but regardless of the mechanism, DPD uptake is likely correlated with amyloid burden in ATTR CA patients since impaired echocardiographic strain in the LV was previously shown to correlate with amyloid burden [31].

DPD_mean_ showed higher correlations to echocardiographic strain parameters compared to DPD_max_. These findings, together with a DPD uptake heterogenicity in different regions of the heart, suggest DPD_mean_ to be a better measure of the severity of the disease. However, in our study, DPD_load_ consistently gave a higher correlation with echocardiographic strain as well as statistically significant correlations for biomarkers in comparison to both DPD_mean_ and DPD_max_. Therefore, DPD_load_ appears to be the most preferable parameter to evaluate disease severity.

We have observed a heterogeneity in uptake distribution over the heart. The uptake pattern among patients ranges from a homogenous uptake pattern to a more heterogeneous pattern with additional higher uptake areas. DPD_load_ is an attempt to quantify both types of patients with a single variable, by making a compound measure of both volume and uptake. During the progression of the ATTR-CA disease, both volume and uptake are expected to increase, which is accentuated in the DPD_load_ measure. We speculate that DPD_load_ may be a method more robust to variations in different uptake patterns, and therefore be a more quantitative measure of the extent of the disease. This assumption is supported by DPD_load_ being the only DPD measure showing significant or near-significant correlation with all three biomarkers (Table 4).

It is hard to assess the influence of the partial volume effect (PVE) in data reported in Table 4. In the following example, we removed the influence of wall thickness by looking at the four patients with 18-mm IVS wall thickness. These patients had a large spread in both IVS DPD uptake and IVS strain. For these four patients, we found that IVS strain correlated well with both DPD_max_ (*R*^2^ = 0.70) and DPD_mean_ (*R*^2^ = 0.65). This example demonstrates the direct correlation between DPD uptake and strain with no contribution of PVE. When there is a range of wall thicknesses, we expect the measured DPD uptake from thinner walls to be more underestimated.

For severe ATTR CA patients, we expect both high DPD uptake and small PVE due to thick heart walls. In patients with less severe disease, we expect low DPD uptake, but also due to a thinner wall, a PVE underestimation of the already low uptake. To summarize, there is a strong correlation between the DPD uptake and the disease severity measure by strain, which is partially modulated by the PVE.

Our finding of less DPD_mean_ in the apex compared to DPD_mean_ in the LV lateral wall and IVS mirrors previously established findings of apical sparing patterns demonstrated in echocardiographic strain, which reinforces that this pathophysiological finding accompanies CA [40]. It is possible that the different wall thickness in apex compared to IVS and LV lateral wall in ATTR-CA could influence the quantification of apical sparing. Since max values are considered more robust to resolution effects, we did a comparison using DPD_max_ and DPD_mean_. When quantifying apical sparing as a DPD ratio, Apex/[ average of IVS and LV-lateral-wall], we found that inserting values for either DPD_mean_ or DPD_max_ gave ratios 0.744 and 0.743. Even though we do not imagine that these methods are as robust as this test happened to suggest, we find it probable that observed apical sparing is a real effect and not entirely created by resolution effects.

In our study, we found a moderate relationship between DPD uptake and RV free wall strain. A possible explanation for a lower correlation toward RV might largely be attributed to less accurate strain analysis for RV free wall strain due to a generally inferior echocardiographic quality in RV visualization often caused by a rather thin RV free wall with relatively few speckles. These weaknesses in strain analysis in general reinforce the potential uses of DPD scintigraphy as a complementary functional assessment tool since DPD scintigraphy is not as patient dependent as strain analysis.

On a small subset of patients, a second observer performed manual delineation of anatomical regions and subsequent 42% thresholding. We noted that the DPD uptake for IVS and LV lateral wall was reproducible within a few percent. This is in line with the excellent interobserver reproducibility of DPD SUV measurements reported elsewhere [27]. However, the delineation of the apex was observer-dependent causing a high variability in both apex DPD uptake and volume. It is possible to have a robust delineation protocol in research. In a clinical setting with segmentation performed by multiple nuclear medicine specialists, we will not expect volume measurements of smaller heart regions to be robust. This affects the DPD_load_ which depends on volume. As a remedy to this problem, we suggest to manually segment and threshold the entire left ventricle (LV) as a practical solution to achieve a robust DPD_load_.

The studies used in this study were performed in a clinical setting with a low-dose CT protocol. This implies that there is not enough contrast between heart tissue and blood to allow segmentation of the lumen. In our study, all ATTRv patients displayed heart tissue DPD uptake that could be used to delineate the heart using a thresholding algorithm.

For patients with low DPD uptake, CT heart delineation might be a feasible method to delineate the heart, in lack of obvious DPD uptake. If we assume a DPD uptake where you cannot discern the heart structure, we could in principle use CT, or CE-CT, to delineate a VOI to quantify this DPD uptake. However, it is questionable if a DPD uptake with low signal-to-noise can give a reliable measurement.

### Clinical and research implications

Since quantitative DPD uptake, measured with SPECT/CT, correlates with myocardial strain and cardiac biomarkers, DPD quantification appears to be more reliable for assessment of disease progression than the Perugini score. Similar to our finding, it has been reported several times that quantitative DPD SPECT/CT cannot distinguish between Perugini score 2–3 patients [24–27]. Furthermore, it has been reported that Perugini scores (higher than zero) displayed no correlation with survival [20]. We find it plausible that the reason for the overlap between Perugini scores 2–3 is that it is based on a visual assessment, and therefore is not quantitatively robust. One further indication is that in our study, Perugini scores did not correlate to biomarkers, except for one case (troponin with Perugini 1 relative 3). We find it plausible that an objective DPD measure based on segmentation has a greater potential to be more robust than a visual score.

In clinical routine, quantification of DPD uptake with a thresholded left ventricle segmentation method is easily obtained and should be reported. A prerequisite for this is that the DPD examination is acquired on a SPECT/CT with attenuation correction along with corrections to decay and a known given dose and sensitivity of the camera. Quantitative DPD SPECT/CT is an easy and widely available tool that could be an alternative or complement to echocardiography for assessment of the efficacy of the new therapeutic treatments of ATTR CA [12, 35].

It is too early to definitely state the added value of quantitative DPD SPECT relative to existing biomarkers. We do however believe that quantitative DPD SPECT, together with other known biomarkers, has the potential to add new information to the basic understanding of ATTR disease progression, as well as in the understanding of the mechanisms in treatment response. Research of novel treatments are under development [11, 12], and new imaging techniques are investigated [24, 34, 35, 41]. It is plausible that quantitative DPD might have an important role in the evaluation of such treatments.

In future works, we wish to investigate the time course of the DPD uptake and the biomarkers on the reported cohort, to evaluate the progression of the disease. We hope to be able to further study DPD uptake during experimental treatment. In this study, we would also like to perform a test-retest to assess the reproducibility of DPD quantification. It remains to be shown that DPD quantification of wild-type ATTR, which has the same type A fibril composition, would reproduce our reported results.

### Limitations

Echocardiographic quality was one limiting factor for this study, i.e. RV strain analysis imaging acquisition was not always possible or optimal. ATTR is also a systemic disease, and in several cases, high DPD uptake was noted in lung tissue and pleural tissue. How this prominent extracardiac uptake affects tracer retention in the heart is still unclear [24]. In the design phase of the study, lung, muscle, and skeletal tissue were supposed to be mapped and used as references in the DPD quantification process; but because of a high interpersonal uptake variance in these tissue references, this method for analysis of the uptake was abandoned. Instead of using tissue references, we adapted to quantify DPD uptake adjusted for body weight and radioactive decay. Due to the retrospective study design, troponin T and NT-ProBNP were not always recorded in connection to the DPD scintigraphic examination. All patients had abdominal tissue biopsy but none of them had a myocardial biopsy. Clinical data were recorded from the clinical records connected to the care district of Umeå University Hospital; therefore, some clinical records on comorbidities may have been missed.

Because cardiac amyloid is a slow progressive disease, we do not expect DPD uptake or echocardiographic strain to change significantly during the time interval of the investigation. Cardiac biomarkers can however change rapidly in patients with coronary artery disease, and these patients were therefore excluded. Natural variation in these biomarkers is high and there is no obvious time for a robust measurement [42]. Consider such large fluctuation, we believe that an up to 30-day interval is going to make the biomarker measurements as accurate as a measurement the same day as the DPD scan.

## Conclusion

We have shown that DPD SPECT/CT uptake in ATTRv patients strongly relates to echocardiographic strain in both the LV and RV. In addition, DPD load significantly correlated to both troponin T and log_10_(NT-ProBNP). These findings suggest that DPD SPECT/CT has the potential to be a valuable tool for the assessment of change in cardiac amyloid load, both in the clinic and in research.
